# Supplementary material for: Antioxidative and Immunomodulatory Potential of the Endemic French Guiana Wild Cocoa “Guiana”
Source: Foods. 2021 Mar 3;10(3):522. doi: 10.3390/foods10030522 (PMC8001100; doi:10.3390/foods10030522)
Supplement: Supplementary file 1 [file foods-10-00522-s001.pdf]

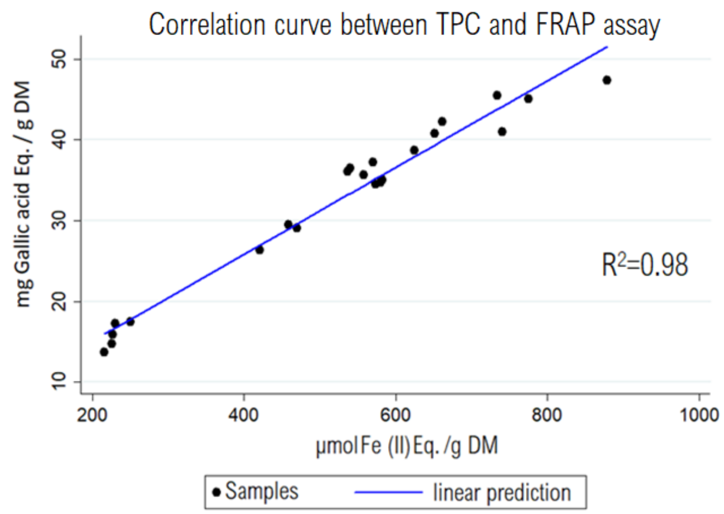

(a)

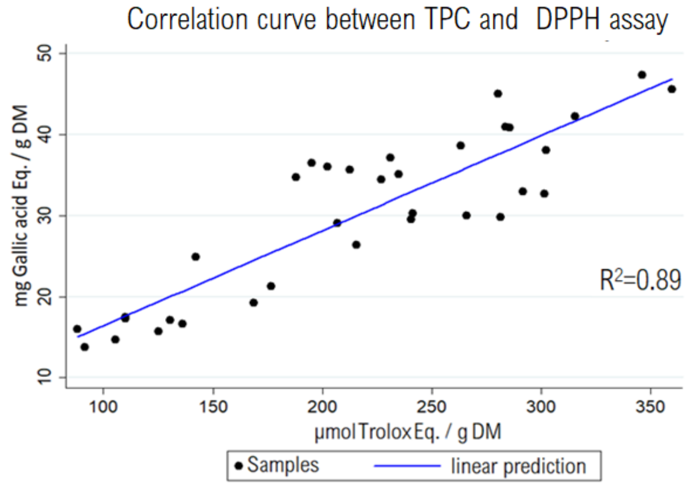

(b)

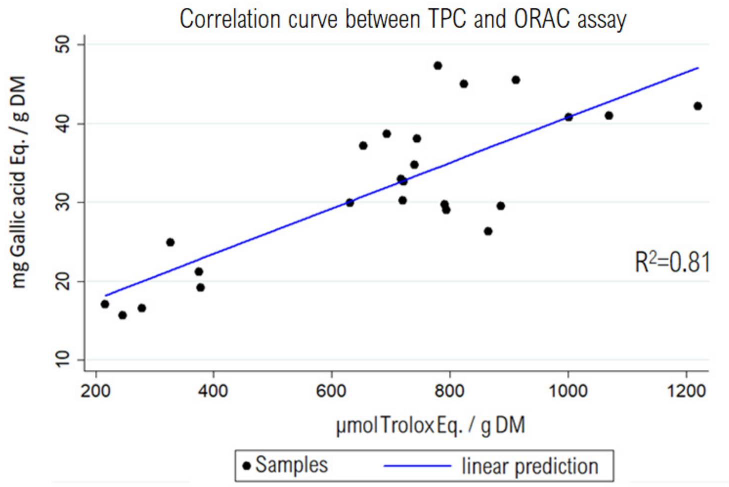

(c)

Figure S1. Correlation curves of Total Polyphenol Contents (TPC) and antioxidant capacities of Guiana and Forastero cocoas (a) FRAP: Ferric Reducing Antioxidant Power assay $p < 0.000$(b) DPPH: 2,2-Diphenyl-1-picrylhydrazyl assay $p < 0.000$ and (c) ORAC: Oxygen Radical Absorbance Capacity assay $p < 0.060$; Gallic acid Eq: gallic acid equivalent; Trolox eq.: Trolox equivalent; Fe (II) eq.: Fe (II) equivalent; DM: dry matter

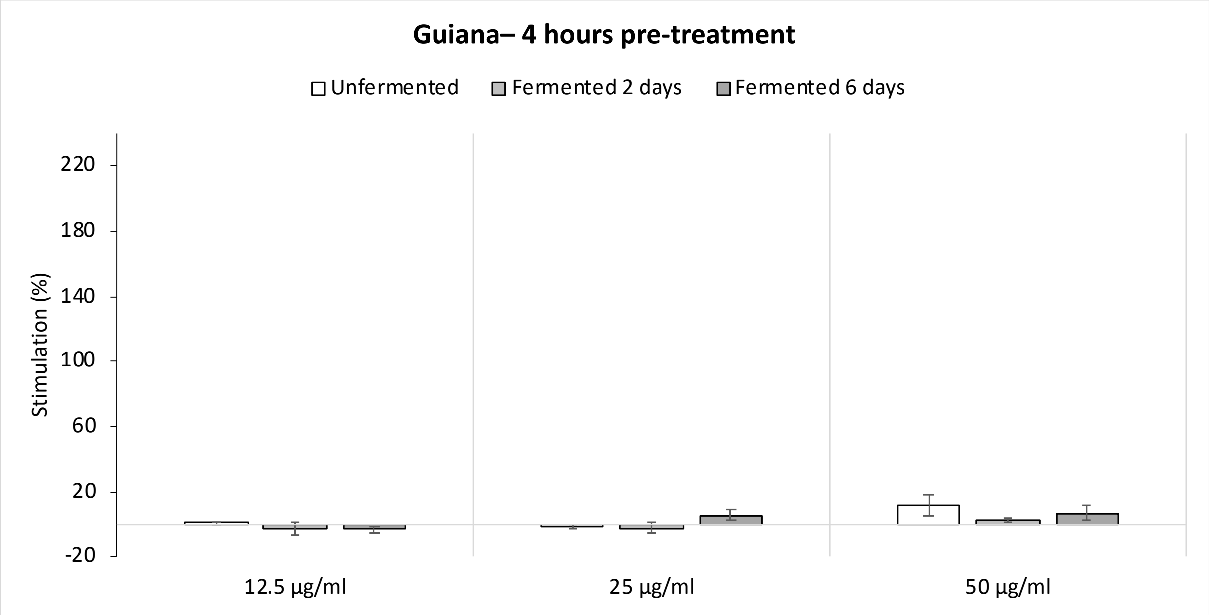

Figure S2: Stimulation production of Tumor necrosis factor alpha (TNF-α) by various concentrations of cocoa extracts (12.5-50 µg/ml) with 4 hours of pre-treatment before inflammatory stimulation with Lipopolysaccharide/ interferon gamma. Results are expressed in Mean ± Sem, (n=4).
